# Plasma metabolomics profiles in Black and White participants of the Adventist Health Study-2 cohort

**DOI:** 10.1186/s12916-023-03101-4

**Published:** 2023-10-31

**Authors:** Fayth M. Butler, Jason Utt, Roy O. Mathew, Carlos A. Casiano, Suzanne Montgomery, Seth A. Wiafe, Johanna W. Lampe, Gary E. Fraser

**Affiliations:** 1 Adventist Health Study, Loma Linda University, Loma Linda, CA USA; 2 Center for Nutrition, Healthy Lifestyle, and Disease Prevention, School of Public Health, Loma Linda University, 24951 Circle Drive, NH2031, Loma Linda, CA 92350 USA; 3 Department of Preventive Medicine, School of Medicine, Loma Linda University, Loma Linda, CA USA; 4 Center for Health Disparities and Molecular Medicine, Loma Linda University School of Medicine, Loma Linda, CA USA; 5 Department of Basic Science, Loma Linda University School of Medicine, Loma Linda, CA USA; 6 Division of Nephrology, Department of Medicine, Loma Linda VA Health Care System, Loma Linda, CA USA; 7 Department of Medicine, School of Medicine, Loma Linda University, Loma Linda, CA USA; 8 School of Behavioral Health, Loma Linda University, Loma Linda, CA 92350 USA; 9 Center for Leadership in Health Systems, School of Public Health, Loma Linda University, Loma Linda, CA USA; 10 Public Health Sciences Division, Fred Hutchinson Cancer Center, Seattle, WA USA

**Keywords:** Metabolomics, Cohort, Dietary pattern, Health disparities, Black Americans, Linear regression, Cardiometabolic, Lipids, Creatine

## Abstract

**Background:**

Black Americans suffer disparities in risk for cardiometabolic and other chronic diseases. Findings from the Adventist Health Study-2 (AHS-2) cohort have shown associations of plant-based dietary patterns and healthy lifestyle factors with prevention of such diseases. Hence, it is likely that racial differences in metabolic profiles correlating with disparities in chronic diseases are explained largely by diet and lifestyle, besides social determinants of health.

**Methods:**

Untargeted plasma metabolomics screening was performed on plasma samples from 350 participants of the AHS-2, including 171 Black and 179 White participants, using ultrahigh-performance liquid chromatography-tandem mass spectrometry (UPLC-MS/MS) and a global platform of 892 metabolites. Differences in metabolites or biochemical subclasses by race were analyzed using linear regression, considering various models adjusted for known confounders, dietary and/or other lifestyle behaviors, social vulnerability, and psychosocial stress. The Storey permutation approach was used to adjust for false discovery at FDR < 0.05.

**Results:**

Linear regression revealed differential abundance of over 40% of individual metabolites or biochemical subclasses when comparing Black with White participants after adjustment for false discovery (FDR < 0.05), with the vast majority showing lower abundance in Blacks. Associations were not appreciably altered with adjustment for dietary patterns and socioeconomic or psychosocial stress. Metabolite subclasses showing consistently lower abundance in Black participants included various lipids, such as lysophospholipids, phosphatidylethanolamines, monoacylglycerols, diacylglycerols, and long-chain monounsaturated fatty acids, among other subclasses or lipid categories. Among all biochemical subclasses, creatine metabolism exclusively showed higher abundance in Black participants, although among metabolites within this subclass, only creatine showed differential abundance after adjustment for glomerular filtration rate. Notable metabolites in higher abundance in Black participants included methyl and propyl paraben sulfates, piperine metabolites, and a considerable proportion of acetylated amino acids, including many previously found associated with glomerular filtration rate.

**Conclusions:**

Differences in metabolic profiles were evident when comparing Black and White participants of the AHS-2 cohort. These differences are likely attributed in part to dietary behaviors not adequately explained by dietary pattern covariates, besides other environmental or genetic factors. Alterations in these metabolites and associated subclasses may have implications for the prevention of chronic diseases in Black Americans.

**Supplementary Information:**

The online version contains supplementary material available at 10.1186/s12916-023-03101-4.

## Background

Black or African Americans suffer persistent disparities in cardiovascular and metabolic diseases, particularly higher morbidity and mortality from these diseases, in spite of downward trends in the general US population. For example, while national mortality rates for heart disease and stroke have declined 61% and 70%, respectively, since 1975, rates for African Americans remain higher by roughly 20% and 40%, respectively [1]. Additionally, rates of hypertension [2, 3] and stroke and peripheral arterial disease have been found to be nearly twice as high in Black compared with White Americans, and Black Americans have higher rates of obesity [2]. Furthermore, Blacks have a disproportionate cancer burden, as evidenced by their higher mortality and low survival compared to other racial/ethnic groups, with a higher risk of death for specific cancers including myeloma, stomach cancer, prostate cancer, endometrial cancer, and breast cancer [4]. Moreover, Blacks are 1.5 to 2 times as likely to develop diabetes as Whites [5, 6] and twice as likely to die from diabetes [7].

Reductions in coronary disease progression and death are largely attributed to improvements in risk factors (smoking, hypertension, physical inactivity) [8, 9]. Dietary patterns or habits may play an important role in the prevention and control of chronic diseases. Epidemiologic and experimental studies have linked diets high in plant-based foods to a number of favorable health outcomes, including lower risk of metabolic syndrome, cardiometabolic diseases, and cancer, whereas diets high in non-fish meats and fatty or refined foods are associated with increased risk of these diseases [10–14]. Findings from the Adventist Health Study (AHS)-2 cohort, particularly, have shown strong inverse associations between a vegetarian dietary pattern and diabetes, metabolic syndrome (including lower triglycerides, glucose, blood pressure, waist circumference, and total and low-density lipoprotein (LDL)-cholesterol), and coronary heart disease [15–19]. This reduction in risk of metabolic syndrome and the favorable outcomes for vegetarians are apparent in Black participants as well [15].

Dietary behaviors may have a profound impact on metabolites. We previously reported distinct metabolic signatures for vegans relative to nonvegetarians, with many of the differentially abundant metabolite subclasses implicated in inflammation-related or cardiometabolic conditions [20]. We have also found higher abundance of anti-inflammatory bioactive compounds and more favorable profiles of fatty acids in vegetarians, especially vegans, relative to non-vegetarians [21]. Racial status and culture may strongly influence dietary patterns and food preferences, consequently impacting overall health and disease susceptibility. Besides dietary behaviors, research has shown that social and environmental determinants of health, as opposed to genetic differences, are critical in propagating the burden of chronic disease disparities in African Americans [22–25]. Blacks are over-represented in lower-wage jobs and more likely to live in lower-income neighborhoods disproportionately higher in poverty compared to White neighborhoods [26, 27]. In these neighborhoods, access to health-promoting resources, and quality health care can be problematic. This socioeconomic disadvantage and social vulnerability may promote psychosocial stress, further contributing to disparities in risk of chronic diseases.

Thus, it is therefore necessary to determine if Black relative to White Americans show differences in metabolic profiles, which may be explained by differences in dietary and behavioral factors, or social and environmental influences, besides genetic factors. In the present study, we used an untargeted metabolomics platform to compare plasma metabolic profiles between Black and White participants of the AHS-2 cohort to determine if differences in metabolites and their associated biochemical subclasses, some with implications for disease susceptibility, could be explained by lifestyle, sociodemographic, or other environmental factors.

## Methods

### Study design/plasma metabolomics profiling

This study included 171 healthy Black (African American, West Indian/Caribbean, African, or other Black) and 179 non-Hispanic White participants from the AHS-2 cohort who previously provided plasma samples in one of three AHS-2 substudies (Calibration, Religion Health Study, Pilot Study) [28–30]. Participants also completed a 204-item food frequency questionnaire (FFQ) at baseline which contained information on dietary habits including consumption of fruits and vegetables, legumes (lentils, soybeans, and other beans), breads and grains, soy foods/drinks/supplements, dairy, eggs, red meats, processed meats, fish, and caffeine/coffee consumption [29]. Participants were subsequently classified by dietary pattern, with vegans never or rarely (< 1/month) consuming animal products including meat, fish, dairy, or eggs; pesco-vegetarians consuming fish at least once per month and other meats < 1/month; and non-vegetarians consuming flesh meats, not only fish ≥ 1/month [29]. The FFQ additionally collected demographic/sociodemographic data including address/zipcode, and other lifestyle behaviors such as exercise, smoking, alcohol drinking, and medication/supplement use.

Plasma samples of 350 study participants were profiled by Metabolon, Inc. (Morrisville, NC) using a global platform (DiscoveryHD4) that provides a comprehensive picture of metabolites within various biochemical classes including amino acid, nucleotide, carbohydrate, lipid, xenobiotic, and microbial classes, further divided into 92 subclasses. Global metabolomics profiling on participant plasma samples was performed in two separate sets (on different days): the first set included samples from 92 participants (pilot), and the second set included a total of 258 samples. Procedures for sample accession and sample preparation including protein precipitation were followed as documented previously [31]. Briefly, samples were prepared using the automated MicroLab STAR® system from Hamilton company, as described previously, and analyzed on four independent ultrahigh-performance liquid chromatography-tandem mass spectrometry (UPLC-MS/MS) platforms. Several quality controls were included — a pooled matrix sample that included a small volume of each experimental sample was used as a technical replicate, water samples were used as process blanks, and a cocktail of quality control internal standards was spiked into every experimental sample to monitor instrument performance. Test samples were randomized across the platform run with QC samples spaced evenly among the injections. Median relative standard deviation (RSD) was calculated for the internal standards added to each sample before injection into the mass spectrometers to determine instrument variability. Process variability was determined by calculating the median RSD for endogenous metabolites present in all of the technical replicates of pooled client samples. The RSD for instrument and process variability was 6% and 8%, respectively. Metabolites were identified by comparison to library entries of purified, authenticated standards or recurrent unknown entities.

A biochemical pathway or “subclass” is annotated for each metabolite within Metabolon’s proprietary reference library of compounds, i.e., lysophospholipids, long-chain monounsaturated fatty acid, long-chain polyunsaturated fatty acid (n3 and n6), xanthine metabolism, creatine metabolism, monoacylglycerol, and diacylglycerol, grouping in accordance with its metabolic function. These subpathways or “subclasses” are grouped into larger/broader pathways or “classes,” i.e., amino acids, lipids, nucleotides, partially characterized molecules (not yet fully characterized), carbohydrates, xenobiotics, drugs, and chemicals.

### Statistical analysis

Raw metabolite values representing mass spectrometry peak densities were median scaled (i.e., for each metabolite, individual values were divided by the median for that metabolite) and log-transformed after first imputing undetectable values with the minimum for each metabolite. Metabolites were excluded from analysis if at least 80% were missing, defined as below the limits of detection, yielding a total of 892 metabolites for statistical analysis. Linear regression was performed on individual metabolites or metabolite subclasses using a combined set of *n* = 350 analytic samples to examine associations of race with log-transformed metabolite abundance, adjusting for relevant covariates or known confounders. Covariates included sex (male vs female), race (Black vs White), age at clinic visit (continuous), batch (continuous), body mass index (BMI, continuous), study (pilot vs non-pilot), substudy (Calibration, Religion Health Study, Pilot), education (high school, some college, college graduate), dietary pattern (vegan, pesco-vegetarian, non-vegetarian), exercise (minutes/week), coffee (ounces/day), use of medications for hypertension or cholesterol or use of aspirin/NSAIDS (yes/no), smoking and alcohol drinking (never, former, current), social vulnerability index (SVI, continuous), psychosocial stress (ordinal), and prevalent chronic diseases including cardiovascular disease, cancer, or diabetes (yes/no).

The Center for Disease Control and Prevention Social Vulnerability Index (SVI) was created by the Agency for Toxic Substances and Disease Registry’s (ATSDR) Geospatial Research, Analysis & Services Program [32], and determined from geocoded addresses for participants in this study. The SVI describes the relative vulnerability of US Census tracts (county subdivisions for which the US Census collects data) based on various social variables, which are grouped into four themes: (1) socioeconomic status, (2) household composition and disability, (3) minority status and language, and (4) housing type and transportation. Each tract received an index or ranking (percentile) for each theme, as well as an overall ranking or combined summary theme, which was used in the current study. Psychosocial stress was defined by two dummy variables — one representing growing up in a single-parent household, defined by divorce of parents or death of a mother or father, “parents divorced” and “mother or father died,” and the other representing personal divorce. Psychosocial stress was a cumulative variable, representing the sum of these two variables.

In the linear regression analysis of differentially abundant metabolites, the adjusted mean with 95% confidence intervals was calculated as the log of the adjusted geometric mean for Black and White participants, and subsequently back-transformed for each metabolite. Fold difference representing the difference in these adjusted means comparing Black with White participants was subsequently calculated.

An adapted Storey et al. permutation approach was used to adjust for false discovery [33], and residualized variables for race were permuted as a means of finding the null distribution of the t scores for metabolite abundance [34], thereby retaining covariances between residualized metabolite abundances. An estimate of the proportion of null metabolites allows for an estimate of the FDR, avoiding the over-conservative Benjamini-Hochberg approach. Consequently, metabolites may be selected with low FDR [33, 35].

For analysis of metabolite subclasses,* t*-scores were obtained by averaging the respective component metabolite values within a subclass and then dividing by the standard deviation of this average, accounting for the covariances between the metabolites. The metabolite subclass average was regressed on race and other covariates. As a sensitivity analysis, and to identify metabolites and subclasses consistently associated with race, linear regression models examining these associations were generated considering pilot study and non-pilot participants separately. All analyses were conducted in R version 4.0.2.

Additionally, over-representation analysis was performed for pathway analysis to determine if a pathway was represented more than expected by chance based on the analytical/reference platform of metabolic pathways in KEGG, performing a hypergeometric test using MetaboAnalyst [36]. Importance measures were calculated from the centrality measures (relative betweenness) reflecting the number of shortest paths going through the node) based on pathway topology, with each pathway having a maximum importance of 1.

A random forest classifier was developed to differentiate race according to metabolic profiles. Two testing techniques were implemented to evaluate the robustness of the random forest classifier. One used a 70% training and 30% test set in a cross-validation approach using all samples and all 892 log-transformed metabolites. In this analysis, each of the 50,000 trees learned from a random sample (70%, training set), with the remaining data (30%, test) passed down the tree for class prediction. Mean decreased accuracy was calculated to determine the most influential metabolites, permuting each predictor variable to assess the difference in predictive accuracy. The second approach was a 5-fold cross-validation (also 70% training and 30% test), which was used to evaluate the model. Overall variable importance was calculated based on the goodness of split measures (ascertained by an impurity function) for each variable. Analyses were conducted in R, using randomForest and rpart packages.

## Results

### Baseline characteristics

The current study included 171 Black and 179 White participants distributed between vegan, pesco-vegetarian, and non-vegetarian diet groups (Table 1). White participants were on average slightly older in years than Blacks (60.4 vs 57.3; *p* < 0.01), with significantly higher coffee consumption (2.2 oz/day vs 0.3 oz/day; *p* < 0.001). Blacks had higher SVI ranking (0.65 vs 0.58; *p* = 0.003), and a greater proportion used blood pressure medication (39% vs 23%; *p* = 0.004) and had a history of smoking (*p* = 0.02) and alcohol drinking (*p* = 0.008). There were additional differences in various dietary components, particularly macronutrients (Additional file 1, Supplementary Table 1). Differences in other characteristics (sex, exercise, diet group, education, disease prevalence) were not statistically significant.

**Table 1:** Demographic and lifestyle characteristics of the study population^a,b^

	**Black**	**White**	***P* value**
Participants	171	179	
Sex			0.67
Male	88 (51.5)	87 (48.6)	
Female	83 (48.5)	92 (51.4)	
Age (years)	57.3 (11.7)	60.4 (10.5)	0.009
BMI (kg/m^2^)	27.7 (5.8)	26.6 (5.5)	0.07
Exercise (min/week)	88.8 (99.5)	92.5 (101.1)	0.74
Diet group			0.84
Vegan	63 (36.8)	62 (34.6)	
Pesco-vegetarian	50 (29.2)	51 (28.5)	
Nonvegetarian	58 (33.9)	66 (36.9)	
Coffee (oz/day)	0.3 (1.0)	2.2 (6.7)	< 0.001
Social vulnerability index (percentile)	0.65 (0.23)	0.58 (0.22)	0.003
Education			0.18
High school	20 (11.8)	23 (15.7)	
Some college	76 (45.0)	63 (37.5)	
College graduate	73 (43.2)	92 (54.8)	
Medication use (yes/no)			
Blood pressure	55 (39)	42 (23.3)	0.004
Cholesterol	23 (16)	32 (17.8)	0.84
Aspirin	42 (29.8)	61 (33.9)	0.51
Smoking			
Ever	43 (25.6)	27 (15.2)	
Never	125 (74.4)	151 (84.8)	0.02
Alcohol			
Current	19 (11.4)	12 (6.7)	0.008
Former	62 (37.3)	46 (25.6)	
Never	85 (51.2)	121 (67.6)	
Prevalent disease			
Cardiovascular	6 (3.5)	6 (3.3)	1
Diabetes	14 (8.2)	10 (5.6)	0.41
Cancer	13 (7.6)	14 (7.8)	1
Psychosocial stress			0.07
No	145	164	
Yes	26	15	

^a^Values presented as *n* (%) or mean (SD)

^b^Missing values: smoking, *n* = 3 (Black), *n* = 1 (White); alcohol *n* = 5 (Black), *n* = 1 (White); education, *n* = 2 (Black), *n* = 1 (White)

### Linear regression analysis of individual metabolites

In order to obtain a more comprehensive understanding of racial disparities contributing to metabolic differences, we generated a number of statistical models to compare metabolomics profiles between Black and White participants. In the simplest model adjusted for age, sex, race, and study, there were 418 differentially abundant metabolites (Tables 2 and 3, Additional file 1, Supplementary Tables 2 and 3). In the most complete model adjusted additionally for various lifestyle behaviors and environmental stressors (BMI, dietary pattern, exercise, smoking, alcohol drinking, coffee consumption, medication use, diabetes, education, SVI, psychosocial stress, and chronic conditions), there were 404 differentially abundant metabolites, with 56 higher and 348 lower in Blacks. Metabolites most notably higher in Black participants included those of the xenobiotics class, such as the methyl/propyl-parabens, propyl- and methyl-4-hydroxybenzoate sulfate within the benzoate metabolism subclass, and various food component/plant metabolites (Table 4). These metabolites in higher abundance overlapped largely with those in the simpler models (Table 2; Additional file 1, Supplementary Table 2), and the most significant metabolites (< 1.0E−3) were retained. Other compounds with the greatest fold difference increases (above 2-fold) or greatest statistical differences (< 4E−04) in Black relative to White participants included various food component/plant metabolites, particularly the piperine metabolites (derivatives of black pepper), 3-carboxy-4-methyl-5-propyl-2-furanpropanoate (CMPF), some acetylated amino acids (and putative uremic toxins), a tocopherol metabolite, bile acid, chemical/drug, and tobacco metabolite (Tables 2 and 4).

**Table 2:** Metabolites (top 40 of 73) present at higher abundance in Black relative to White participants at FDR < 0.05 in simplest model^a,b^

**Metabolite**	**Fold difference**	**Subclass**	**Major class**	**FDR (Storey)**
Propyl 4-hydroxybenzoate sulfate	7.57	Benzoate metabolism	Xenobiotics	< 3.6E−04
Methyl-4-hydroxybenzoate sulfate	4.56	Benzoate metabolism	Xenobiotics	< 3.6E−04
Piperine	3.32	Food component/plant	Xenobiotics	5.2E−04
3-Carboxy-4-methyl-5-propyl-2-furanpropanoate (cmpf)	3.23	Fatty acid, dicarboxylate	Lipid	8.8E−04
Umbelliferone sulfate	2.95	Food component/plant	Xenobiotics	< 3.6E−04
Sulfate of piperine metabolite c18h21no3 (1)	2.79	Food component/plant	Xenobiotics	< 3.6E−04
n-Acetyl-1-methylhistidine	2.78	Histidine metabolism	Amino acid	< 3.6E−04
Hydroxy-cmpf	2.73	Fatty acid, dicarboxylate	Lipid	1.9E−03
Thymol sulfate	2.72	Food component/plant	Xenobiotics	9.9E−04
Sulfate of piperine metabolite c16h19no3 (3)	2.71	Food component/plant	Xenobiotics	< 3.6E−04
Sulfate of piperine metabolite c16h19no3 (2)	2.66	Food component/plant	Xenobiotics	< 3.6E−04
Sulfate of piperine metabolite c18h21no3 (3)	2.58	Food component/plant	Xenobiotics	< 3.6E−04
2-Hydroxyfluorene sulfate	2.49	Tobacco metabolite	Xenobiotics	< 3.6E−04
2-Naphthol sulfate	2.49	Chemical	Xenobiotics	< 3.6E−04
n-Acetylalliin	2.31	Food component/plant	Xenobiotics	1.5E−03
Gamma-cehc sulfate	2.23	Tocopherol metabolism	Cofactors and vitamins	< 3.6E−04
n-Acetylcitrulline	2.18	Urea cycle; arginine and proline metabolism	Amino acid	< 3.6E−04
2-Piperidinone	2.04	Food component/plant	Xenobiotics	< 3.6E−04
Glucuronide of piperine metabolite c17h21no3 (4)	2.02	Food component/plant	Xenobiotics	1.0E−02
Glucuronide of piperine metabolite c17h21no3 (3)	1.93	Food component/plant	Xenobiotics	1.1E−02
Glucuronide of piperine metabolite c17h21no3 (5)	1.91	Food component/plant	Xenobiotics	1.0E−02
Taurodeoxycholic acid 3-sulfate	1.89	Secondary bile acid metabolism	Lipid	5.8E−03
Glutarate (c5-dc)	1.59	Fatty acid, dicarboxylate	Lipid	3.6E−04
Perfluorooctanesulfonate (pfos)	1.51	Chemical	Xenobiotics	1.0E−02
n-Acetylarginine	1.49	Urea cycle; arginine and proline metabolism	Amino acid	< 3.6E−04
Serotonin	1.48	Tryptophan metabolism	Amino acid	4.8E−02
n-Acetylglutamine	1.45	Glutamate metabolism	Amino acid	< 3.6E−04
Indoleacetylglutamine	1.44	Tryptophan metabolism	Amino acid	3.1E−02
n-Acetylasparagine	1.42	Alanine and aspartate metabolism	Amino acid	< 3.6E−04
Hexanoylglutamine	1.42	Fatty acid metabolism (acyl glutamine)	Lipid	1.3E−02
1-Stearoyl-2-docosahexaenoyl-gpc (18:0/22:6)	1.41	Phosphatidylcholine (PC)	Lipid	3.8E−04
n-Acetyl-2-aminooctanoate	1.40	Fatty acid, amino	Lipid	2.5E−03
6-Oxopiperidine-2-carboxylate	1.40	Lysine metabolism	Amino acid	9.8E−03
Taurocholenate sulfate	1.38	Secondary bile acid metabolism	Lipid	3.5E−03
Campesterol	1.37	Sterol	Lipid	3.1E−02
Carotene diol (3)	1.36	Vitamin A metabolism	Cofactors and vitamins	5.0E−03
Arginine	1.35	Urea cycle; arginine and proline metabolism	Amino acid	< 3.6E−04
Cis-4-decenoylcarnitine (c10:1)	1.34	Fatty acid metabolism (acyl carnitine, monounsaturated)	Lipid	5.0E−04
Picolinate	1.33	Tryptophan metabolism	Amino acid	8.5E−03
1-(1-Enyl-stearoyl)-2-arachidonoyl-gpe (p-18:0/20:4)	1.33	Plasmalogen	Lipid	2.3E−03

^a^Fold difference represents the ratio of geometric means of raw metabolite values of Black relative to White participants

^b^Adjusted for age, sex, batch, study, and substudy

**Table 3:** Metabolites (top 40 of 345) present at lower abundance in Black relative to White participants at FDR < 0.05 in simplest model^a,b^

**Metabolite**	**Fold difference**	**Subclass**	**Major class**	**FDR (Storey)**
Theobromine	0.26	Xanthine metabolism	Xenobiotics	9.2E−04
n-Methylpipecolate	0.28	Bacterial/fungal	Xenobiotics	4.5E−03
Caffeine	0.29	Xanthine metabolism	Xenobiotics	3.6E−03
n6-methyllysine	0.30	Lysine metabolism	Amino acid	2.3E−03
3-Methylxanthine	0.32	Xanthine metabolism	Xenobiotics	1.2E−04
5-Acetylamino-6-amino-3-methyluracil	0.34	Xanthine metabolism	Xenobiotics	1.1E−03
Tryptophan betaine	0.35	Tryptophan metabolism	Amino acid	3.8E−04
7-Methylxanthine	0.37	Xanthine metabolism	Xenobiotics	1.6E−04
1-Methylxanthine	0.40	Xanthine metabolism	Xenobiotics	1.9E−04
1,7-Dimethylurate	0.40	Xanthine metabolism	Xenobiotics	1.2E−03
2-Palmitoleoyl-gpc (16:1)	0.41	Lysophospholipid	Lipid	8.2E−05
Theophylline	0.44	Xanthine metabolism	Xenobiotics	5.3E−03
Galactonate	0.45	Fructose, mannose, and galactose metabolism	Carbohydrate	2.0E−04
5alpha-androstan-3beta,17alpha-diol disulfate	0.46	Androgenic steroids	Lipid	8.9E−05
2-Hydroxyacetaminophen sulfate	0.47	Drug — analgesics, anesthetics	Xenobiotics	2.3E−02
Quinate	0.47	Food component/plant	Xenobiotics	5.1E−03
1,3-Dimethylurate	0.47	Xanthine metabolism	Xenobiotics	7.8E−04
Paraxanthine	0.48	Xanthine metabolism	Xenobiotics	1.4E−02
Palmitoyl-linoleoyl-glycerol (16:0/18:2) [1]	0.49	Diacylglycerol	Lipid	9.8E−05
3-Aminoisobutyrate	0.49	Pyrimidine metabolism, thymine containing	Nucleotide	5.7E−04
Oleoyl-linoleoyl-glycerol (18:1/18:2) [2]	0.49	Diacylglycerol	Lipid	6.5E−04
Alpha-cehc sulfate	0.49	Tocopherol metabolism	Cofactors and vitamins	6.9E−04
Metabolonic lactone sulfate	0.50	Partially characterized molecules	Partially characterized	2.2E−04
1-Palmitoleoylglycerol (16:1)	0.50	Monoacylglycerol	Lipid	1.8E−04
2-Aminophenol sulfate	0.51	Food component/plant	Xenobiotics	2.9E−04
2-Palmitoleoylglycerol (16:1)	0.51	Monoacylglycerol	Lipid	1.1E−04
4-Ethylcatechol sulfate	0.52	Benzoate metabolism	Xenobiotics	1.1E−03
1-Methylurate	0.52	Xanthine metabolism	Xenobiotics	1.8E−04
n6,n6-dimethyllysine	0.52	Lysine metabolism	Amino acid	1.5E−03
Vanillic acid glycine	0.53	Food component/plant	Xenobiotics	1.9E−04
Palmitoloelycholine	0.53	Fatty acid metabolism (acyl choline)	Lipid	4.1E−04
Linoleoyl-linolenoyl-glycerol (18:2/18:3) [2]	0.54	Diacylglycerol	Lipid	9.2E−05
n-Delta-acetylornithine	0.54	Urea cycle; arginine and proline metabolism	Amino acid	2.3E−04
1,3,7-Trimethylurate	0.54	Xanthine metabolism	Xenobiotics	2.1E−03
Myristoleate (14:1n5)	0.55	Long-chain monounsaturated fatty acid	Lipid	1.3E−02
3-Methylglutarylcarnitine (2)	0.55	Leucine, isoleucine, and valine metabolism	Amino acid	2.7E−04
Cholate	0.55	Primary bile acid metabolism	Lipid	1.9E−03
Indolebutyrate	0.55	Tryptophan metabolism	Amino acid	2.1E−04
Oleoyl-linoleoyl-glycerol (18:1/18:2) [1]	0.56	Diacylglycerol	Lipid	7.5E−04
Ethylmalonate	0.57	Leucine, isoleucine, and valine metabolism	Amino acid	9.0E−04

^a^Fold difference represents the ratio of geometric means of raw metabolite values of Black relative to White participants

^b^Adjusted for age, sex, batch, study, and substudy

Compound represents structural isomer in the Metabolon spectral library

**Table 4:** Metabolites present at higher abundance in Black relative to White participants at FDR < 0.05 in the most completely adjusted model^a,b^

**Metabolite**	**Fold difference**	**Subclass**	**Major class**	**FDR (Storey)**
Propyl 4-hydroxybenzoate sulfate	7.25	Benzoate metabolism	Xenobiotics	< 7.6E−05
Methyl-4-hydroxybenzoate sulfate	4.29	Benzoate metabolism	Xenobiotics	< 7.6E−05
Piperine	3.19	Food component/plant	Xenobiotics	3.8E−03
n-Acetylalliin	2.80	Food component/plant	Xenobiotics	2.1E−03
Sulfate of piperine metabolite c16h19no3 (3)	2.67	Food component/plant	Xenobiotics	1.1E−03
Sulfate of piperine metabolite c18h21no3 (1)	2.66	Food component/plant	Xenobiotics	1.2E−03
Sulfate of piperine metabolite c18h21no3 (3)	2.51	Food component/plant	Xenobiotics	8.7E−04
Umbelliferone sulfate	2.49	Food component/plant	Xenobiotics	4.8E−03
n-Acetyl-1-methylhistidine	2.44	Histidine metabolism	Amino acid	< 7.6E−05
3-Carboxy-4-methyl-5-propyl-2-furanpropanoate (cmpf)	2.43	Fatty acid, dicarboxylate	Lipid	5.9E−03
Sulfate of piperine metabolite c16h19no3 (2)	2.41	Food component/plant	Xenobiotics	1.0E−03
2-Hydroxyfluorene sulfate	2.33	Tobacco metabolite	Xenobiotics	< 7.6E−05
2-Naphthol sulfate	2.32	Chemical	Xenobiotics	< 7.6E−05
Hydroxy-cmpf	2.17	Fatty acid, dicarboxylate	Lipid	1.0E−02
n-Acetylcitrulline	2.16	Urea cycle; arginine and proline metabolism	Amino acid	< 7.6E−05
Thymol sulfate	2.14	Food component/plant	Xenobiotics	5.0E−02
Genistein sulfate	2.13	Food component/plant	Xenobiotics	3.7E−02
Gamma-cehc sulfate	2.11	Tocopherol metabolism	Cofactors and vitamins	1.1E−03
Glycohyocholate	1.89	Secondary bile acid metabolism	Lipid	5.8E−03
2-Piperidinone	1.87	Food component/plant	Xenobiotics	5.9E−03
Taurodeoxycholic acid 3-sulfate	1.86	Secondary bile acid metabolism	Lipid	1.5E−02
Tauro-beta-muricholate	1.72	Primary bile acid metabolism	Lipid	2.6E−02
Glutarate (c5-dc)	1.54	Fatty acid, dicarboxylate	Lipid	6.8E−03
n-Acetylglutamine	1.49	Glutamate metabolism	Amino acid	< 7.6E−05
Taurocholenate sulfate	1.47	Secondary bile acid metabolism	Lipid	6.2E−03
Carotene diol (3)	1.47	Vitamin A metabolism	Cofactors and vitamins	5.0E−03
Spermidine	1.45	Polyamine metabolism	Amino acid	2.0E−02
n-Acetylarginine	1.45	Urea cycle; arginine and proline metabolism	Amino acid	9.6E−04
n-Acetyl-2-aminooctanoate	1.43	Fatty acid, amino	Lipid	1.2E−02
n-Acetylasparagine	1.38	Alanine and aspartate metabolism	Amino acid	2.0E−03
Cis-4-decenoylcarnitine (c10:1)	1.34	Fatty acid metabolism (acyl carnitine, monounsaturated)	Lipid	6.5E−03
Homoarginine	1.33	Urea cycle; arginine and proline metabolism	Amino acid	1.0E−02
1-(1-Enyl-stearoyl)-2-arachidonoyl-gpe (p-18:0/20:4)	1.29	Plasmalogen	Lipid	1.4E−02
Sphingomyelin (d18:1/20:1, d18:2/20:0)	1.26	Sphingomyelins	Lipid	< 7.6E−05
Arginine	1.26	Urea cycle; arginine and proline metabolism	Amino acid	6.8E−03
Creatine	1.26	Creatine metabolism	Amino acid	9.7E−03
Carotene diol (2)	1.26	Vitamin A metabolism	Cofactors and vitamins	3.7E−02
3-Methoxytyrosine	1.25	Tyrosine metabolism	Amino acid	1.6E−02
Sphingomyelin (d18:2/24:2)	1.25	Sphingomyelins	Lipid	9.6E−04
Guanidinoacetate	1.25	Creatine metabolism	Amino acid	6.9E−03
Succinylcarnitine (c4-dc)	1.25	TCA cycle	Energy	3.5E−02
2′-o-methyluridine	1.24	Pyrimidine metabolism, uracil containing	Nucleotide	1.5E−02
2-Hydroxyglutarate	1.23	Fatty acid, dicarboxylate	Lipid	9.3E−03
1-(1-Enyl-palmitoyl)-2-linoleoyl-gpc (p-16:0/18:2)	1.22	Plasmalogen	Lipid	1.2E−03
1-(1-Enyl-palmitoyl)-2-arachidonoyl-gpe (p-16:0/20:4)	1.22	Plasmalogen	Lipid	3.5E−02
Sphingomyelin (d18:2/18:1)	1.21	Sphingomyelins	Lipid	2.7E−02
Sphingomyelin (d18:1/20:2, d18:2/20:1, d16:1/22:2)	1.20	Sphingomyelins	Lipid	2.0E−02
1-(1-Enyl-palmitoyl)-2-arachidonoyl-gpc (p-16:0/20:4)	1.19	Plasmalogen	Lipid	6.2E−03
Sphingomyelin (d18:1/22:2, d18:2/22:1, d16:1/24:2)	1.18	Sphingomyelins	Lipid	6.3E−03
1-(1-Enyl-palmitoyl)-2-palmitoyl-gpc (p-16:0/16:0)	1.17	Plasmalogen	Lipid	2.0E−03
Gamma-glutamylthreonine	1.15	Gamma-glutamyl amino acid	Peptide	3.7E−02
5-Methylthioribose	1.15	Methionine, cysteine, SAM, and taurine metabolism	Amino acid	9.4E−03
1-(1-Enyl-palmitoyl)-2-oleoyl-gpc (p-16:0/18:1)	1.15	Plasmalogen	Lipid	2.1E−02
Sphingomyelin (d18:1/17:0, d17:1/18:0, d19:1/16:0)	1.13	Sphingomyelins	Lipid	2.7E−02
Betaine	1.12	Glycine, serine, and threonine metabolism	Amino acid	3.5E−02
Creatinine	1.10	Creatine metabolism	Amino acid	2.2E−02

^a^Fold difference represents the ratio of geometric means of raw metabolite values of Black relative to White participants

^b^Adjustment for age, sex, batch, substudy, dietary pattern, education, social vulnerability index, exercise, BMI, smoking, alcohol drinking, coffee, medication use, and chronic disease (cardiovascular disease, diabetes, cancer, hypertension)

Among metabolites showing the lowest abundance in Black relative to White participants was the microbial metabolite n-methylpipecolate, along with lysine metabolites (n6-methyllysine, n6,n6-dimethyllysine), various xanthine metabolites, and tryptophan betaine, represented in both the simplest and fullest models (Tables 3 and 5; Additional file 1, Supplementary Table 4). Various types of fatty acids and phospholipids showed the greatest statistical significance among metabolites inversely associated with Black race. Comparing the fully adjusted with simplest model, there was some attenuation of representation of acyl carnitines and secondary bile acids, among other compounds, and greater representation of leucine, isoleucine, and valine metabolites, as well as long-chain polyunsaturated fatty acids, and glycine, serine, threonine metabolites in the fully adjusted model. Overall, results from the fully adjusted model were very similar to other less complex models (Additional file 1, Supplementary Tables 5–8), where across these models at least 58 metabolites were higher, and 345 lower in Black participants, with the most notable difference being attenuation of representation of acyl carnitines in the fullest relative to the simpler models.

**Table 5:** Top 40 (of 348) metabolites present at lower abundance in Black relative to White participants at FDR < 0.05 in the most completely adjusted model^a,b^

**Metabolite**	**Fold difference**	**Subclass**	**Major class**	**FDR (Storey)**
n-Methylpipecolate	0.27	Bacterial/fungal	Xenobiotics	4.8E−03
n6-methyllysine	0.31	Lysine metabolism	Amino acid	2.4E−03
Theobromine	0.33	Xanthine metabolism	Xenobiotics	1.9E−03
Tryptophan betaine	0.35	Tryptophan metabolism	Amino acid	1.8E−04
Myristoleate (14:1n5)	0.36	Long-chain monounsaturated fatty acid	Lipid	1.2E−03
3-Methylxanthine	0.37	Xanthine metabolism	Xenobiotics	2.5E−04
Palmitoyl-linoleoyl-glycerol (16:0/18:2) [1]	0.38	Diacylglycerol	Lipid	2.3E−04
5-Acetylamino-6-amino-3-methyluracil	0.39	Xanthine metabolism	Xenobiotics	2.7E−03
2-Palmitoleoylglycerol (16:1)	0.40	Monoacylglycerol	Lipid	3.0E−04
Metabolonic lactone sulfate	0.41	Partially characterized	Partially characterized	4.8E−04
Alpha-cehc sulfate	0.41	Tocopherol metabolism	Cofactors and vitamins	5.3E−04
Galactonate	0.42	Fructose, mannose, and galactose metabolism	Carbohydrate	4.0E−04
2-Hydroxyacetaminophen sulfate	0.42	Drug — analgesics, anesthetics	Xenobiotics	2.9E−02
7-Methylxanthine	0.43	Xanthine metabolism	Xenobiotics	1.5E−03
1-Palmitoleoylglycerol (16:1)	0.44	Monoacylglycerol	Lipid	2.2E−04
Oleoyl-linoleoyl-glycerol (18:1/18:2) [2]	0.45	Diacylglycerol	Lipid	1.2E−03
Caffeine	0.45	Xanthine metabolism	Xenobiotics	8.2E−03
Linoleoyl-linolenoyl-glycerol (18:2/18:3) [2]	0.45	Diacylglycerol	Lipid	1.6E−04
1,7-Dimethylurate	0.46	Xanthine metabolism	Xenobiotics	2.6E−03
1-Methylxanthine	0.47	Xanthine metabolism	Xenobiotics	1.2E−03
Docosatrienoate (22:3n6)	0.48	Long-chain polyunsaturated fatty acid (n3 and n6)	Lipid	2.1E−04
3-Methylglutarylcarnitine (2)	0.48	Leucine, isoleucine, and valine metabolism	Amino acid	4.0E−04
Palmitoleate (16:1n7)	0.49	Long-chain monounsaturated fatty acid	Lipid	2.0E−04
2-Palmitoleoyl-gpc (16:1)	0.49	Lysophospholipid	Lipid	4.0E−03
9-Hydroxystearate	0.49	Fatty acid, monohydroxy	Lipid	8.1E−05
Linoleoyl-linoleoyl-glycerol (18:2/18:2) [2]	0.50	Diacylglycerol	Lipid	8.2E−05
5alpha-androstan-3beta,17alpha-diol disulfate	0.50	Androgenic steroids	Lipid	1.3E−03
Vanillic acid glycine	0.51	Food component/plant	Xenobiotics	9.6E−04
3-Aminoisobutyrate	0.51	Pyrimidine metabolism, thymine containing	Nucleotide	4.3E−04
Ceramide (d16:1/24:1, d18:1/22:1)	0.51	Ceramides	Lipid	1.9E−04
Palmitoloelycholine	0.52	Fatty acid metabolism (acyl choline)	Lipid	2.8E−04
2-Aminophenol sulfate	0.52	Food component/plant	Xenobiotics	2.0E−03
Oleoyl-linoleoyl-glycerol (18:1/18:2) [1]	0.53	Diacylglycerol	Lipid	1.6E−03
1-Palmitoyl-2-palmitoleoyl-gpc (16:0/16:1)	0.53	Phosphatidylcholine (PC)	Lipid	9.6E−04
Palmitoyl-linoleoyl-glycerol (16:0/18:2) [2]	0.53	Diacylglycerol	Lipid	2.1E−04
n6,n6-dimethyllysine	0.53	Lysine metabolism	Amino acid	8.0E−04
Cystathionine	0.54	Methionine, cysteine, SAM, and taurine metabolism	Amino acid	1.4E−03
5alpha-androstan-3alpha,17beta-diol monosulfate (1)	0.54	Androgenic steroids	Lipid	4.0E−04

^a^Fold difference represents the ratio of geometric means of raw metabolite values of Black relative to White participants

^b^Adjusted for age, sex, batch, substudy, dietary pattern, education, social vulnerability index, exercise, BMI, coffee, medication use, and chronic disease (cardiovascular disease, diabetes, cancer, hypertension)

Compound represents structural isomer in the Metabolon spectral library

A sensitivity analysis considering only the 258 participants not included in the pilot study [20] showed overall fewer significant metabolites (*n* = 296), although 95% (281) of metabolites significant in the analysis considering these participants were also significant considering the full dataset (Additional file 1, Supplementary Table 9), including metabolites showing the greatest fold differences or statistical significance. A similar analysis of pilot participants revealed 268 differential metabolites, and again, the majority of these were also differential in the analysis considering the full cohort (75%), and nearly half overlapped with the non-pilot study participants (Additional file 1, Supplementary Tables 10 and 11).

### Linear regression analysis of metabolite subclasses

Besides examining associations of race with individual metabolites, we also conducted an analysis of metabolite subclasses by averaging metabolites across their respective biochemical subclasses. Results from the fully adjusted model including adjustment for lifestyle/behavioral variables and environmental stress revealed a total of 38 differentially abundant metabolite subclasses of a total of 92 queried subclasses, comparing the two racial groups (Table 6). Creatine metabolism exclusively showed higher abundance in Blacks (fold change = 1.20). Besides creatine metabolism, subclasses with the strongest significance included long-chain monounsaturated fatty acid, pyrimidine metabolism, ceramides, and several phospho- and glycero-lipid subclasses (notably lysophospholipid, monoacylglycerol, phosphatidylethanolamine, phosphatidylinositol, glycerolipid metabolism, diacylglycerol, monoacylglycerol, phosphatidylethanolamine, glycerolipid metabolism), leucine, isoleucine, and valine metabolism, and lysine metabolism, all showing lower abundance in Blacks. Results from less complex models including only basic covariates (age, sex, substudy, and batch excluding BMI) or basic covariates in addition to dietary pattern, exercise, and/or environmental stress variables showed a similar representation of the most significant subclasses with 44–54 differential metabolic subclasses detected, all represented at lower abundance, with the exception of creatine metabolism (Additional file 1, Supplementary Tables 12–14).

**Table 6:** Metabolite subclasses associated with race (Black vs White) at FDR < 0.05 in the most completely adjusted model^a,b^

**Subclass**	**Fold difference (95% CI)**	***n* total metabolites**	***n* significant metabolites**	***n* > 1**	***n* < 1**	**FDR**
Creatine metabolism	1.20 (1.11, 1.29)	3	3	3	0	< 9.0E−05
Long-chain monounsaturated fatty acid	0.61 (0.49, 0.75)	7	7	0	7	9.5E−05
Pyrimidine metabolism, thymine containing	0.73 (0.63, 0.83)	2	1	0	1	1.2E−04
Ceramides	0.74 (0.65, 0.85)	9	8	0	8	1.4E−04
Lysophospholipid	0.75 (0.67, 0.84)	29	24	0	24	1.6E−04
Monoacylglycerol	0.65 (0.54, 0.79)	16	13	0	13	1.6E−04
Phosphatidylethanolamine (PE)	0.70 (0.60, 0.83)	12	10	0	10	1.8E−04
Phosphatidylinositol (PI)	0.75 (0.67, 0.83)	6	6	0	6	2.4E−04
Glycerolipid metabolism	0.77 (0.68, 0.87)	2	2	0	2	2.6E−04
Tryptophan metabolism	0.84 (0.77, 0.92)	20	11	0	11	3.3E−04
Diacylglycerol	0.57 (0.47, 0.68)	11	10	0	10	4.7E−04
Pyrimidine metabolism, orotate containing	0.79 (0.70, 0.90)	3	2	0	2	7.1E−04
Long-chain polyunsaturated fatty acid (n3 and n6)	0.72 (0.61, 0.86)	17	14	0	14	7.7E−04
Leucine, isoleucine, and valine metabolism	0.87 (0.80, 0.94)	28	15	0	15	7.8E−04
Lysine metabolism	0.83 (0.75, 0.92)	16	6	0	6	9.5E−04
Fatty acid metabolism (acyl choline)	0.72 (0.60, 0.87)	9	8	0	8	9.8E−04
Xanthine metabolism	0.49 (0.32, 0.74)	13	12	0	12	1.5E−03
Ascorbate and aldarate metabolism	0.84 (0.75, 0.93)	6	5	0	5	2.2E−03
Phosphatidylcholine (PC)	0.85 (0.77, 0.94)	19	10	0	10	2.4E−03
Fatty acid, dihydroxy	0.85 (0.76, 0.94)	5	3	0	3	2.9E−03
Fructose, mannose, and galactose metabolism	0.79 (0.68, 0.92)	4	1	0	1	3.3E−03
Guanidino and acetamido metabolism	0.73 (0.60, 0.89)	2	1	0	1	3.4E−03
Pentose metabolism	0.85 (0.76, 0.95)	6	4	0	4	4.7E−03
Long-chain saturated fatty acid	0.82 (0.72, 0.95)	7	5	0	5	7.0E−03
Dihydroceramides	0.79 (0.66, 0.94)	2	2	0	2	7.4E−03
Pyrimidine metabolism, cytidine containing	0.84 (0.74, 0.96)	5	2	0	2	1.3E−02
Eicosanoid	0.75 (0.59, 0.95)	3	2	0	2	1.7E−02
Chemical	0.87 (0.78, 0.98)	20	9	1	8	1.9E−02
Fatty Acid, monohydroxy	0.90 (0.82, 0.98)	19	5	0	5	2.0E−02
Androgenic steroids	0.80 (0.66, 0.97)	21	8	0	8	2.1E−02
Pantothenate and CoA metabolism	0.78 (0.63, 0.98)	2	1	0	1	3.0E−02
Gamma-glutamyl amino acid	0.93 (0.86, 1.00)	15	9	1	8	3.4E−02
Nicotinate and nicotinamide metabolism	0.86 (0.75, 0.99)	5	2	0	2	3.6E−02
Sphingosines	0.88 (0.78, 1.00)	2	1	0	1	3.6E−02
Purine metabolism, adenine containing	0.94 (0.88, 1.00)	5	2	0	2	4.0E−02
Fatty acid metabolism (acyl carnitine, dicarboxylate)	0.86 (0.73, 1.01)	4	2	0	2	4.6E−02
Sphingolipid synthesis	0.89 (0.79, 1.00)	3	2	0	2	4.6E−02
Phospholipid metabolism	0.92 (0.84, 1.00)	6	2	0	2	4.7E−02

^a^Linear regression analysis based on composite t-statistics derived by dividing by the standard deviation of the averaged, log-transformed metabolites

^b^Adjustment for age, sex, batch, substudy, dietary pattern, education, social vulnerability index, exercise, BMI, smoking, alcohol drinking, coffee, medication use, and chronic conditions (CVD, diabetes, hypertension, cancer)

For many of the differentially represented subclasses, the vast majority of component metabolites were significantly differential (names of component metabolites in Additional file 1, Supplementary Table 15), and showed lower abundance in Blacks, as determined from linear regression analysis of individual metabolites. Examples are the long-chain monounsaturated fatty acid, monoacylglycerol, phosphatidylethanolamine, ceramides, lysophospholipid, diacylglycerol, long-chain polyunsaturated fatty acid, and xanthine metabolism subclasses (Table 6). Several of the top subclasses were replicated, both in analyses excluding or including only pilot participants, with many in common between both sub-cohorts. (Additional file 1, Supplementary Table 16). Pathway analysis of metabolic pathways in KEGG revealed an over-representation of biosynthesis of unsaturated fatty acids pathway (FDR = 0.02), consistent with results from subclass analyses (Additional file 1, Supplementary Table 17).

In light of the finding of several differentially abundant subclasses of lipids, we performed additional analyses to adjust for dietary fat, including mono-, poly-, and saturated fat consumption. Results from the linear regression analysis of individual metabolites were overall similar to those from other models (Additional file 1, Supplementary Table 18). However, far fewer lipid/fatty acid and other subclasses were statistically differential after adjustment for dietary fat, although several were retained, including long-chain monounsaturated fatty acids among others (Additional file 1, Supplementary Table 19). Additionally, given the relevance of several differentially abundant metabolites or biochemical subclasses related to kidney function, namely, creatine metabolism and acetylated amino acids, we also compared metabolic profiles among a subset of individuals with plasma creatinine measurements (mg/dL) adjusting for estimated glomerular filtration rate (eGFR). Adjustment for eGFR (mL/min/m^2^) resulted in far fewer metabolites showing higher abundance in Blacks, notably many acetylated amino acids, which were no longer significant, although creatine was still present in higher abundance. Interestingly, the inclusion of eGFR in analyses of differential abundance of biochemical subclasses yielded a higher number of differential subclasses, with some showing stronger statistical significance, most notably, lysine metabolism (Additional file 1, Supplementary Tables 20–23). Creatine metabolism, however, was no longer significant after adjustment for eGFR.

### Random forest

**Fig. 1:**
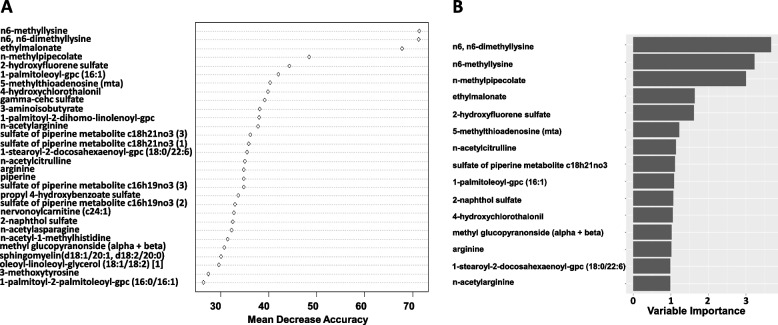
Random forest analysis showing **A** mean decrease accuracy representing an average decrease in accuracy after permutation of each respective metabolite and **B** importance plot using 5-fold cross-validation, classifying Black and White participants

Random forest analysis was performed to identify metabolic profiles differentiating race, reasoning that metabolites showing the greatest importance in distinguishing race might be more reflective of biological differences. Random forest classification showed a predictive accuracy in the test set of 81.3% and revealed the greatest discriminating potential for n6,n6-dimethyllysine, n6-methyllysine, n-methylpipecolate, ethylmalonate, and 2-hydroxyfluorene sulfate (Fig. 1A). These metabolites were among those found to be most differential in the linear regression analysis. Random forest was also performed using 5-fold cross-validation, with a predictive accuracy of 87.9%, and overlap of the top 5 metabolites showing the greatest variable importance (Fig. 1B), thereby providing evidence of the robustness of the random forest classifier and validating the discriminating ability of the most influential metabolites.

## Discussion

Differences in metabolite abundance may account for racial disparities in susceptibility to chronic diseases [37–39]. Using an untargeted metabolomics platform, we investigated differences in plasma metabolites with roles in various biological pathways with relevance to chronic diseases. We noted differences in a number of xenobiotic and lipid metabolite subclasses — particularly lysophospholipids, mono- and diacylglycerols, ceramides, and related lipids.

We hypothesized that inter-related lifestyle and socioeconomic factors largely contribute to racial differences in metabolic profiles. However, expanded models adjusting for these variables did not appreciably alter results, neither did adjustment for lifestyle (dietary pattern, exercise, etc.) and social stress variables, as the number of differential metabolites was not diminished, although differences in some xenobiotic metabolites are likely explained by differences in lower caffeine consumption (as many xanthine metabolites are caffeine derivatives), and lower medication use (i.e., drugs/analgesics) by Black participants. Additionally, the differences we noted in consumption of select foods or nutrients may partially explain differences in lipids and other metabolites not evident with adjustment for dietary pattern alone. For example, two of the most differentially abundant or discriminating metabolites from linear regression or random forest analyses, n-methylpipecolate and tryptophan betaine (lower in Blacks) have been associated with consumption of certain types of legumes [40–42], and legumes were among foods consumed at higher amounts among Black participants.

Dietary habits and food preferences could have a major impact on the types/distribution on lipid metabolites found to be differential between Black and White participants, for example, phospholipids or diacylglycerols, besides triglycerides in plasma (again lower in Blacks). Synthesis of diacylglycerols and phospholipids is heavily influenced by diet. Indeed, we noted differences in dietary intakes of saturated and unsaturated fatty acids, which helped explain differences in biochemical subclasses or pathways relevant to lipid biosynthesis or metabolism. Fatty acids from the diet diffuse in micelles from the gut lumen into enterocytes and are converted to phosphatidic acid, and subsequently diacylglycerols or phospholipids, and later secreted as vesicles into plasma in chylomicrons or very-low-density lipoprotein, and transported to other tissues [43]. Lysophoshospholipids are intermediates of other phospholipids, generated by enzymatic activity resulting in the removal of an acyl group. Variations in the diet (consumption of oleic or palmitic acids, and related oils) could mean variations in phospholipid profiles, as well as the fatty acids incorporated into the C1 and C2 positions of diacylglycerols, which are continually in flux [44]. Hence differences in some of these lipid types reflect differences in food preferences, which are culturally influenced. Accordingly, Black Americans within AHS-2 were shown to have higher consumption of certain types of carbohydrates and “soul” or Caribbean foods such as macaroni and cheese, red/pinto beans, rice and beans, black-eyed peas, okra, and other foods [45], which could contribute to differences in lipid metabolism. This is consistent with the higher consumption of carbohydrates in Black participants in the current study.

A high carbohydrate diet might increase de novo lipogenesis and consequently phospholipids or triglycerides, as glucose impacts this process by providing acetyl-coA, and can induce expression or production of lipogenic genes or enzymes [46]. Induction of lipogenic genes is also evident in the context of obesity. Lysophospholipid metabolism is also altered with obesity, and in turn impacts obesity-related diseases [47]. Besides differences in diet or obesity, the other possible explanation for differences in phospholipids and other fatty acids relates to ethnic differences in triglycerides. Black Americans have been found to have lower triglycerides in spite of higher rates of several cardiometabolic diseases, termed “the triglyceride paradox” [48], although the reason is not clear. It is not clear from the current study how levels of plasma triglycerides differed between the two racial groups. But there were lower levels of long-chain fatty acids and monoacylglycerols, which are products of triglyceride digestion by pancreatic lipase. Thus, it is possible that lower levels of triglycerides correlate with lower products of triglyceride digestion (i.e., lower monoacylglycerols and monounsaturated/long-chain fatty acids) in Blacks. As glycerol and glycerol-3-phosphate (comprising the glycerophospholipids subclass) are precursors for phospholipids and di- and triacylglycerols, it might be expected that these would be significantly lower in Blacks.

No lipid metabolite subclasses showed higher abundance in Black participants. There was, however, a proportionately greater representation of sphingomyelins and plasmalogens in Black participants when considering individual metabolites. Both plasmalogens and sphingomyelins have important roles in cell structure and signaling. While their pathophysiological roles are not completely clear, dysregulation may be associated with neurodegenerative disorders, cancer, cardiovascular disease, diabetes, and other metabolic diseases [49, 50] which could explain higher rates of these diseases in Blacks.

Lysine and leucine metabolism showed significant and consistent differences comparing Black and White participants. Products of leucine breakdown such as 3-methylglutaconate and ethylmalonate, among others, have been previously associated with single nucleotide polymorphisms (SNPs) [51], which could partly explain the differences. Ethylmalonate is a branched fatty acid that has been associated with a protein-altering variant in acyl-CoA dehydrogenase (ACAD), which may affect fatty acid oxidation [51, 52]. This is consistent with the differences observed in fatty acid and lipid metabolism in the current study, as leucine and lysine catabolism both yield acetyl CoA to regulate fatty acid synthesis. It was interesting that methylated lysines, particularly, showed the strongest ability to differentiate Black and White participants. These metabolites are especially interesting because of their potential involvement in regulation of gene expression. For instance, histone lysine methylation may be associated with activation or silencing of genes depending on position [53, 54], and these metabolites could have been present in plasma as a result of histone degradation. It is also possible that these lysine residues are nonhistone derived. Nonhistone lysine methylation has implications for oncogenesis and cancer progression [55]. As methylation of lysine is regulated by lysine methyltransferase, it would be interesting to determine in future studies if such enzymes show differential activity in Black and White Americans in the context of both health and disease.

The creatine metabolism subclass was exclusively higher in Black participants, with all three component metabolites statistically higher in the fully adjusted model. Higher creatine was the top metabolite associated positively with estimated glomerular filtration rate (eGFR) in African American participants of the Atherosclerosis Risk in Communities (ARIC) study without chronic kidney disease at baseline [56]. In the current study, race was associated with creatine metabolism after adjustment for social, demographic, and environmental factors, and creatine (though not creatine metabolism) showed differential abundance even after adjustment for eGFR in a sub-analysis. Our finding of higher abundance of creatine or the creatine metabolism subclass in Blacks may highlight clinically relevant racial distinctions, as creatinine is the principal determinant of eGFR [57]. Because even healthy Black or African Americans have shown higher levels of creatinine, various equations have been generated to calculate eGFR with adjustment for race. But the use of such equations is controversial as they often yield higher estimates of GFR in Blacks, leading to a greater number of undiagnosed cases. Recently, a new race-free calculation of eGFR was developed with either creatinine or another filtration marker called cystatin-C [58]. Given the apparently complex role of race in creatine metabolism, metabolomics analysis will likely provide increased discriminatory power to GFR estimation in the future.

The higher abundance of creatine-related metabolites was paralleled by higher abundance of arginine, a precursor for creatine that has been found to be higher in other individuals of African descent [59]. Even more interestingly, though not directly related to creatine metabolism, a number of n-acetylated metabolites, which have relevance to kidney function, showed significant differences in the current study, though in both directions. A number of these metabolites have been found inversely associated with eGFR in African Americans [56, 60], many also associated with SNPs more common in African Americans [51, 60], and particularly mapping to loci related to acetylated amino acids, consequently impacting enzyme-metabolite interactions. Ten of 12 recently identified metabolites with novel validated inverse associations with creatinine glomerular filtration rate in novel subpathways (as discovered among participants of the ARIC and Bogalusa Heart Study (BHS) cohorts), were significantly lower in Black participants in the current study, including some metabolites showing greatest fold changes or discriminating ability, such as n-methylpipecolate, and 3-amino-isobutyrate, among others. Consequently, the observed racial differences in abundance of acetylated amino acids which are putative uremic solutes, and particularly those in higher abundance in Black participants, may have important implications for kidney health disparities as Blacks present with significantly higher kidney function diseases.

Metabolites of methyl- and propyl- parabens, common preservatives in foods, cosmetics (hair, skin, etc.), and medications, were markedly higher (~ 6-fold) in Blacks. African or Black Americans have shown higher levels of parabens in urine in other studies [61], likely attributable to hair products [62]. Particularly hair products have been found to contain endocrine-disrupting chemicals and have been linked with breast cancer (due to parabens or other chemicals) [13, 62–67], and are used more commonly by Black women [67, 62, 68]. Though parabens are generally considered safe, there are some questions about toxicity given some reports of oxidative DNA damage upon light irradiation in dermal tissues [69]. Our findings warrant further investigation of these metabolites in studies with Black participants and biospecimens.

The minimal impact of other lifestyle factors, and socioeconomic or psychosocial stressors (which were not notably different between Black and White participants in this study) on metabolic profiles might be explained by the overall healthier lifestyles among Black AHS-2 participants when compared to the general population. AHS-2 participants have healthier lifestyles, diminishing some of the social disparities that exist between Black and White Americans, and translating into better health outcomes for Black participants. The AHS-2 cohort consists of a large proportion of individuals following vegetarian or plant-based diets, with very little tobacco smoking or alcohol drinking, consistent with the religious doctrine. Thus these participants are more alike in such areas that would otherwise contribute to confounding, yet with variety in dietary habits/patterns and other lifestyle factors. While these are unique features or strengths of this cohort, there are inevitable limitations in comparability with other populations. An external cohort of non-Adventists will have notably different lifestyles and behaviors which impact health outcomes and biological pathways impacting disease. AHS-2 participants, including Blacks, have lower overall cancer incidence and lower all-cause and/or cancer mortality compared to the National Longitudinal Mortality Study (NLMS) population and its Surveillance, Epidemiology, and End Results (SEER) substudy, representing US census populations [70]. The religious engagement and church activity also may favorably impact health and mortality. Such findings highlight the relevance of healthy lifestyles, behaviors, and social experiences (healthy diets, absence of smoking, alcohol drinking, religion, management of stress) in controlling racial disparities. Consequently, there are limitations in terms of external validity.

It is not clear how the differential metabolic profiles might relate to susceptibility to metabolic diseases, and it is challenging to disentangle the biological and social effects in explaining racial differences, which may be inherently intertwined. For example, social stress may interact with biological factors and particularly genetic or epigenetic factors that influence metabolite abundance. The metabolic profile is attributable to various biological and environmental factors, i.e., diet, genetic polymorphisms, the gut microbial community, physical activity, and stress, among others. The statistical models generated were not comprehensive with regard to covariates, and there is a likelihood of residual confounding, particularly in light of the strong relationship between diet and metabolites. However, we have attempted to examine the contributions of lifestyle and social factors by generating and comparing models of varying complexity. Hence, the observed racial differences in metabolic profiles should be interpreted with caution. Nonetheless, many of the differentially abundant lipid subclasses have important physiological roles, and consequently implications for health and disease. Lysophospholipids and other signaling lipids have roles in cardiometabolic and neurological health, and inflammatory responses [71, 72], besides obesity [47]. Besides dietary components/nutrients, it is possible that the lower abundance of certain metabolites reflects differences in body composition that were unaddressed with adjustment for BMI. The potential contribution of genetic polymorphisms also should not be ignored, which might impact kidney health or function and disease risk. Interestingly, metabolomic lactone sulfate which was markedly lower in Black participants, has been associated with a polymorphism in a CYP3A gene and poor cardiometabolic health. An association of such biomarkers with a lower risk of cardiometabolic and other diseases in Black Americans may highlight, all the more, racial disparities in the progression of chronic diseases, and the critical contributions of social determinants of health.

This study has notable strengths, including the approximately equal numbers of Black and White participants with many lifestyle similarities, and comprehensive data collected on study participants, allowing for considerable model complexity (inclusion of data on social vulnerability, dietary patterns, and other lifestyle behaviors and environmental stress). One issue, however, is that the Black participants in this cohort may not reflect the general population as far as socioeconomic or -cultural characteristics, which could be seen as a strength or a limitation. The Black AHS-2 participants overall have a higher level of education and experience less socioeconomic disadvantage. Another limitation is the somewhat small sample size for the current study precluding more comprehensive comparisons considering aspects of diet or stratified analyses, although there was more than enough power to detect statistical differences in plasma metabolites by race, and we were able to repeat analyses comparing two sub-samples.

## Conclusions

Black and White participants show distinct metabolic profiles, most notably differences in phospholipid metabolism and related lipid subclasses, besides creatine and lysine metabolism, and metabolites with relevance to kidney function. Differences in some of these metabolites and subclasses are likely a reflection of unique dietary behaviors, not simply dietary patterns, while other differences may be attributed to genetic influences. Many differences in xenobiotic metabolites probably relate to lifestyle choices, such as the use of medications and caffeinated beverages. Whether attributed to dietary, genetic, or other factors, these differential metabolic profiles may have implications for cardiometabolic and renal health in Blacks, driving various health disparities outcomes.

### Supplementary Information

**Additional file 1: Supplementary Table 1.** Dietary intakes of study participants. **Supplementary Table 2.** All metabolites present at higher abundance in Black relative to White participants at FDR < 0.05 in simplest model. **Supplementary Table 3.** All metabolites present at lower abundance in Black relative to White participants at FDR < 0.05 in simplest model. **Supplementary Table 4.** Metabolites (*n* = 348) present at lower abundance in Black relative to White participants at FDR < 0.05. **Supplementary Table 5.** Metabolites present at higher abundance in Black relative to White participants at FDR < 0.05 (with additional adjustment for BMI and dietary pattern). **Supplementary Table 6.** Metabolites present at lower abundance in Black relative to White participants at FDR < 0.05 (with additional adjustment for BMI and dietary pattern). **Supplementary Table 7.** Metabolites present at higher abundance in Black relative to White participants at FDR < 0.05 (with additional adjustment for BMI, dietary pattern, exercise, social vulnerability index, and psychosocial stress). **Supplementary Table 8.** Metabolites present at lower abundance in Black relative to White participants at FDR < 0.05 (with additional adjustment for BMI, dietary pattern, exercise, social vulnerability index, and psychosocial stress). **Supplementary Table 9.** Metabolites present at lower abundance in Black relative to White participants at FDR < 0.05 excluding pilot study participants. **Supplementary Table 10.** Metabolites present at lower abundance in Black relative to White participants at FDR < 0.05 in pilot study participants. **Supplementary Table 11.** Differentially abundant metabolites in common between analyses with pilot (*n* = 93) and non-pilot (*n* = 258) study participants at FDR<0.05. **Supplementary Table 12.** Metabolite subclasses associated with race (Black vs White) at FDR in simplest model < 0.05. **Supplementary Table 13.** Metabolite subclasses associated with race (Black vs White) at FDR < 0.05 (adjusted additionally for BMI and dietary pattern). **Supplementary Table 14.** Metabolite subclasses associated with race (Black vs White) at FDR < 0.05 (adjusted additionally for BMI, dietary pattern, social vulnerability index, and psychosocial stress). **Supplementary Table 15.** Names of statistically significant component metabolites (based on linear regression analysis) contained within each subclass differentially abundant at FDR < 0.05. **Supplementary Table 16.** Differentially abundant subclasses in common between analyses with pilot (*n* = 93) and non-pilot (*n* = 258) study participants at FDR<0.05. **Supplementary Table 17.** Results from pathway analysis of metabolic pathways over-represented comparing Black and White participants (at *P* ≤ 0.5). **Supplementary Table 18.** Metabolites differentially abundant in Black relative to White participants at FDR < 0.05 (adjusted additionally for dietary pattern, dietary fat (polyunsaturated, monounsaturated, and saturated), education, social vulnerability index, exercise, BMI, coffee, medication use, chronic disease cardiovascular disease, diabetes, hypertension). **Supplementary Table 19.** Metabolite subclasses associated with race (Black vs White) at FDR < 0.05 (adjusted additionally for BMI, dietary pattern, social vulnerability index, psychosocial stress, and dietary fat). **Supplementary Table 20.** Metabolites present at higher abundance in Black relative to White participants at FDR < 0.05 in *n* = 140 study participants with plasma creatinine measurements (and adjustment for eGFR). **Supplementary Table 21.** Metabolites present at higher abundance in Black relative to White participants at FDR < 0.05 in *n* = 140 study participants with plasma creatinine measurements. **Supplementary Table 22.** Metabolite subclasses associated with race (Black vs White) in subset of study participants with plasma creatinine (and adjustment for eGFR) at FDR < 0.05. **Supplementary Table 23.** Metabolite subclasses associated with race (Black vs White) in subset of study participants with plasma creatinine at FDR < 0.05.

## Data Availability

The datasets used and/or analyzed during the current study are not publicly available due to data use restrictions but will be available from the corresponding author on reasonable request.

## Abbreviations

ACAD: Acyl-CoA dehydrogenase
AHS-2: Adventist Health Study-2
ATSDR: Agency for Toxic Substances and Disease Registry
BMI: Body mass index
NLMS: National Longitudinal Mortality Study population
SEER: Surveillance, Epidemiology, and End Results
SNP: Single nucleotide polymorphism
UPLC-MS/MS: Ultrahigh-performance liquid chromatography-tandem mass spectroscopy
